# Non-human Papillomavirus Cervical Mucinous Adenocarcinoma in a Phenotypic Male with Congenital Adrenal Hyperplasia

**DOI:** 10.7759/cureus.3607

**Published:** 2018-11-18

**Authors:** Jennifer Heim, Elizabeth Dickson, Melissa AA Geller, Colleen Rivard

**Affiliations:** 1 Obstetrics and Gynecology, Midwest Women's Healthcare Specialists, Kansas City, USA; 2 Obstetrics and Gynecology, Aurora Health, Milwaukee, USA; 3 Obstetrics and Gynecology, University of Minnesota, Minneapolis, USA

**Keywords:** cervical cancer, congenital adrenal hyperplasia

## Abstract

A majority of cervical cancers are caused by human papillomavirus (HPV); however, HPV-negative cervical cancers exist and, though rare, are more aggressive. No prior reports examine HPV-negative cancer of the cervix in a female pseudohermaphrodite with congenital adrenal hyperplasia (CAH). This is a case of a 78-year-old phenotypic male with hypospadias and absent testicles who presented with urinary retention and urosepsis. He was diagnosed with a pelvic mass on imaging and with a female mosaic karyotype (45,X/47,XXX/46 XX). He was taken to the operating room and found to have a rare form of HPV-negative cervical cancer: gastric-type adenocarcinoma (GAS). This study examines the presentation, management, and outcome of a GAS cervical cancer in a patient with a known lack of HPV exposure secondary to the unique anatomy of female pseudohermaphrodism.

## Introduction

Cervical cancer remains the third most common cancer among women worldwide. The majority of cervical cancer requires the persistence of an oncogenic genital human papillomavirus (HPV). This ubiquitous virus is transmitted sexually and most often is cleared in the immunocompetent individual. However, a small percentage of high-risk HPV-infected individuals develop cervical intraepithelial neoplasia (CIN) that can progress from low-grade to high-grade and eventually cervical cancer in the setting of necessary cofactors. Though rare and accounting for a small percentage, there is a subset of cervical cancers that are HPV-negative [1]. These cancers tend to be more aggressive and of a less common histology. To the best of our knowledge, this is the first report of a non-HPV related cervical cancer in a genotypic female virilized phenotypic male secondary to congenital adrenal hyperplasia (CAH).

## Case presentation

**Figure 1:**
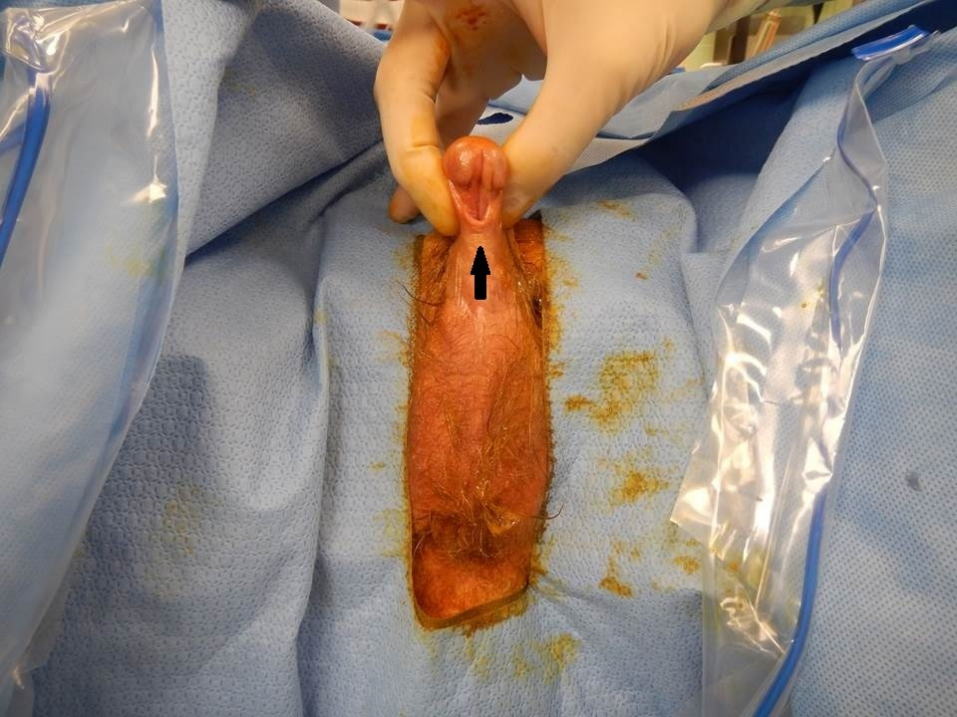
Patient's external genitalia External genitalia demonstrating hypospadias (arrow) and absent testicles.

A 78-year-old phenotypic male presented with acute onset of urinary retention and urosepsis. The patient reported a long history of hesitancy and frequency with recent abdominal pain and hematuria for one-week duration. Attempts to place a transurethral catheter failed, and a supra-pubic catheter was inserted. The patient was treated for urosepsis with intravenous ciprofloxacin and piperacillin/tazobactam in the intensive care unit and showed clinical improvement. His past medical history was significant for infertility as well as an unknown abdominal surgical procedure as a very young child. The patient reported puberty at age 14, normal sexual activity and regular erections up to two months prior to presentation. The urology service attempted to perform bedside cystoscopy, which demonstrated a normal distal urethra and obliteration of the entire lumen on entrance into the bulbar urethra. A computed tomography (CT) scan was performed showing bilateral adrenal masses consistent with myolipomas unchanged in size from a 2001 CT scan and an enlarged pelvic mass arising from what was thought to be the adnexa compressing the urinary bladder. On physical examination, the patient was noted to have hypospadias and absent testicles (Figure 1). The patient had undergone an unknown procedure as a child but did not have any further information about his medical history and no further follow-up was ever done per the patient report.

Based on these clinical and radiographic findings the patient underwent karyotype testing and was found to have mosaic 45,X/47,XXX/46 XX karyotype during his admission. Based on the abnormal laboratory values, the endocrinology service was consulted, and he was diagnosed with CAH due to 11-hydroxylase deficiency. His lab values confirmed the diagnosis of 11-hydroxylase deficiency showing elevated estradiol, testosterone, androstenedione, dihydroepiandrosterone-sulfate (DHEAS), 11-deoxycortisol, 17-hydroxyprogesterone, and adrenocorticotropic hormone (ACTH) using the reference ranges for female patients (Table 1).

**Table 1:** Patient laboratory values

Laboratory Test	Patient Value	Reference Range
Follicle stimulating hormone (FSH)	<0.3 IU/L	1.4 – 18.1 IU/L
Luteinizing hormone (LH)	<0.1 IU/L	3.1 – 34.6 IU/L
Estradiol	68 pg/mL	<15 pg/mL
Testosterone Total Free	443 ng/dL 7.5 ng/dL	8 – 60 ng/dL 0.3 – 1.9 ng/dL
Androstenedione	27 ng/mL	0.23 – 0.89 ng/mL
Dehydroepiandrosterone (DHEA)	4.1 ng/mL	0.63 – 4.7 ng/mL
DHEA sulfate	>1000 μg/dL	80 – 560 μg/dL
Cortisol	9.7 μg/dL	4-22 μg/dL
11-Deoxycortisol	59 ng/dL	<49 ng/dL
17-OH progesterone	9676 ng/dL	20 – 190 ng/dL
17-OH pregnenolone	270 ng/dL	<442 ng/dL
Aldosterone	25.8 ng/dL	4 – 31 ng/dL
Deoxycorticosterone	9.6 ng/dL	2 – 19 ng/dL
Adrenal corticotropin (ACTH)	112 pg/mL	10 – 47 pg/mL

**Figure 2:**
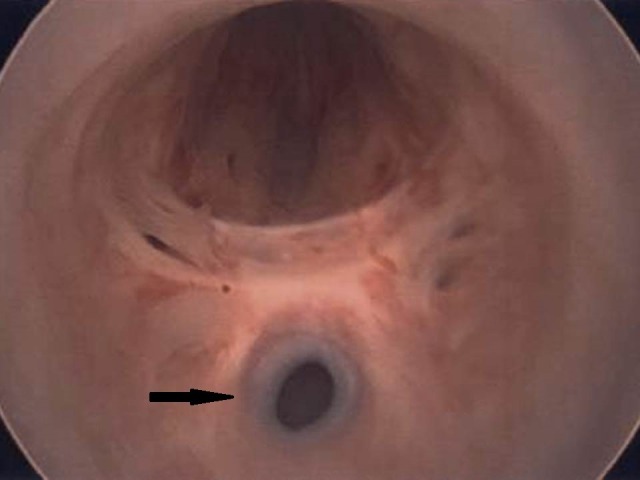
Cystoscopy view View of the blind vaginal opening (arrow) off the patient's posterior urethra on cystoscopy.

Given the pelvic mass, he was taken to the operating room for a combined procedure by the urology and gynecologic oncology teams. The procedure was initiated with an adrenal biopsy followed by cystoscopy. The cystoscopy revealed a posterior passage off the urethra toward a remnant vagina, cervix, and uterus (Figure 2).

An exploratory laparotomy, total abdominal hysterectomy, bilateral salpingo-oophorectomy, omental biopsy, and bilateral pelvic lymph node dissection was performed. The intraoperative findings included an enlarged uterus densely adherent to the bowel and omentum. The bilateral ovaries appeared normal. The cervix was replaced by a tumor extending to the serosa and protruding into the vagina but not involving the blind narrow vaginal pouch. Pelvic lymph nodes were enlarged but the remainder of the abdominal and pelvic surveys were negative for obvious tumor involvement. The pathology from this procedure showed benign adrenal tissue and mucinous intestinal-type adenocarcinoma of the endocervix with the absence of HPV. His final diagnosis was Fédération Internationale de Gynécologie et d'Obstétrique (FIGO) stage IIIB mucinous adenocarcinoma of the endocervix with positive pelvic lymph nodes. The staining of the tumor was positive for epithelial membrane antigen (EMA), cytokeratin seven (CK7), p63 (patchy), estrogen receptor (patchy, weak nuclear staining), monoclonal carcinoembryonic antigen (CEA) (focal), and mucicarmine. The tumor was negative for p16, cluster of differentiation 10 (CD10), calretinin and uroplakin.

The patient recovered as expected and was discharged home. He received adjuvant chemotherapy and radiation with weekly cisplatin and whole pelvic external beam radiation at 45 Gray (Gy) followed by an external tumor bed boost to 59 Gy, given the inability to treat with standard vaginal brachytherapy following whole pelvic radiation.

One month following completion of chemo radiation, the patient was noted to have new uptake near the bladder on a positron emission tomography (PET) scan. The patient was offered chemotherapy but declined further treatment and presented one month later with ST segment elevation myocardial infarction (STEMI) and acute kidney injury (AKI). He would have required dialysis, which he did not wish to pursue. He was discharged to hospice and died three days later.

## Discussion

CAH is an autosomal recessive disease that generally results from the functional absence of one of the enzymes involved in the conversion of cholesterol to cortisol. The most common form of CAH is caused by 21-hydroxylase deficiency. Both male and female infants with the salt wasting form of 11-hydroxylase deficiency are likely to present in the second or third week of life with hyperkalemia, hyponatremia, and hypovolemia. Male individuals without the salt wasting form can go undiagnosed in infancy and present years later with signs or symptoms of androgen excess. Female infants are more likely to be brought to medical attention due to ambiguous genitalia as a result of in utero exposure to the excess androgens that result from the enzyme deficiency, as was the case with this patient.

In utero, the fetus has both Wolffian and Mullerian ducts. In order for a fetus to rid itself of the internal female reproductive organs, an anti-Mullerian hormone (AMH) must be present. In the case of our patient and other female pseudohermaphrodites, there is a lack of the SRY gene located on the Y chromosome, which leads to the development of testes and subsequent production of AMH by sertoli cells. In the absence of AMH, Mullerian ducts differentiate into internal female reproductive organs. The development of male external and internal genitalia depends on both testosterone and dihydrotestosterone (DHT). In the female fetus with CAH, abnormally high levels of testosterone result in the maintenance and development of the Wolffian duct through local rather than systemic testosterone, which is necessary for development of testes. This explains the lack of testicular development in this case. The urogenital sinus and genital tubercle are virilized in both male and female fetuses in the presence of high levels of DHT. Gynecologic cancers in genotypic females with CAH are rare: the literature reveals only 10 case reports of ovarian steroid cell tumors (OSCTs) associated with CAH. There are no case reports of cervical malignancy specifically associated with CAH. Thus, removing the organs making up the female internal genitalia in an individual being raised male is not strongly supported at this time due to the lack of outcome data.

Not only is a gynecologic cancer in a female pseudohermaphrodite exceedingly rare, the type of cervical cancer diagnosed in this patient is also uncommon. Rather than the more common endocervical type adenocarcinoma, the tumor was identified as mucinous adenocarcinoma with goblet cells otherwise known as mucinous intestinal type adenocarcinoma of the endocervix. In the presence of high-risk HPV, the retinoblastoma protein (Rb)—which normally inhibits the transcription of p16—becomes inactivated by the HPV E7 protein [2-4]. Thus, this lack of p16 staining suggests a lack of high-risk HPV in the cervical neoplasm of our patient. CD10 and calretinin stain positive in the presence of Wolffian remnants. The absence of these two markers in our patient points away from a Wolffian origin and suggests the absence of mesonephric glands in the pathology specimen [5-9]. A negative uroplakin staining pattern was utilized to rule out a tumor of transitional cell origin. The diffusely positive EMA is suggestive of either CIN or cervical neoplasia. The monoclonal CEA stain is generally negative in endometrial adenocarcinomas and positive in endocervical adenocarcinomas. Its positivity in this case lends to a diagnosis of cervical-origin adenocarcinoma. A positive CEA and CK7 point toward intestinal type cells. Finally, the presence of goblet cells and mucicarmine positive staining narrows the diagnosis to a mucinous intestinal-type adenocarcinoma.

Mucinous intestinal-type adenocarcinoma of the cervix is also known as gastric-type adenocarcinoma (GAS) of the cervix and was first given this designation as a subtype of mucinous adenocarcinoma in the World Health Organization (WHO) classification [9]. GAS is unique in that it develops in the absence of high-risk HPV. It is also known to have a poor prognosis. In one review of 53 cases of mucinous adenocarcinoma of the cervix, the survival difference between gastric type and nongastric type was significantly worse for the patients with gastric type (25% versus 75% five-year disease-free survival) [10].

The unique external genital anatomy of this patient needed to be taken into consideration in order to provide the most appropriate treatment to our patient. The patient received the standard cisplatin chemotherapy with extended field radiation therapy at the recommended dose of 40 – 45 Gy. Given the lack of a cavity due to his unique anatomy there was no site for the placement of intracavitary radiation, only external beam radiation was used to give the tumor bed a total dose of 59 Gy in order to accommodate the unique circumstances and provide care as close as possible to the standard of care.

## Conclusions

Most cervical cancers are related to HPV infection. CAH is a congenital disorder that can lead to a genotypically female individual displaying phenotypical male characteristics. This is a rare case of a phenotypic male, genotypic female who presented with mucinous adenocarcinoma of the cervix consistent with a non-HPV related tumor. This is the first ever reported case of its type.
